# Study on a high precision alignment system with dual cameras

**DOI:** 10.1371/journal.pone.0339765

**Published:** 2026-02-20

**Authors:** Chao-Chung Liu, Chih-Chiang Fang, Chao-Shu Liu

**Affiliations:** 1 School of Computer Science and Software, Zhaoqing University, Zhaoqing, China; 2 Department of Mechanical Engineering, ^‌^National Kaohsiung University of Science and Technology, Kaohsiung, Taiwan; Polytechnic Institute of Leiria: Instituto Politecnico de Leiria, PORTUGAL

## Abstract

This study focuses on developing an automatic high-precision alignment system that integrates automated optical inspection with machine vision recognition to replace traditional manual alignment methods. The core aim is to implement an image-based positioning and control system for a precision alignment platform with dual cameras. Various image processing techniques, such as template matching, edge detection, mathematical morphology, and Hough transforms, are used to identify target features. The identified target coordinates are then fed back to the controller, which calculates the necessary compensation for the XXY platform to achieve precise alignment. For motion control, both XYZ and XXY platforms are utilized to perform image-based alignment tasks. Objects are first transported on the XYZ platform, then precisely positioned and aligned on the XXY platform equipped with dual machine visions, which is equivalent to simulate high-precision automation in production lines. The control system is built on an FPGA development platform integrated with a PC-based architecture and embedded the control algorithms, Proportional-Integral-Derivative (PID) control method and Manifold Deformation Design Scheme (MDDS). Experimental results show that MDDS offers superior performance, compared with PID method, including better accuracy, improved trajectory tracking, and greater control stability. Additionally, the image processing system achieves a high precision, with the flexibility to switch targets, enhancing the system’s practical applicability in real-world scenarios. The proposed system is validated for use in automated production lines, demonstrating its high potential for industrial applications that require precise alignment and control.

## 1. Introduction

With the rapid development of precision industries such as semiconductor manufacturing, display panel production, precision electronic assembly, optical component fabrication, and biomedical equipment, the demand for ultra-high alignment accuracy has grown significantly. High-precision alignment platforms have thus become indispensable equipment in modern manufacturing systems, directly determining product quality, efficiency, and yield. However, traditional manual alignment operations, even when assisted by high-magnification optical devices, often suffer from visual fatigue, inconsistent standards, and operator-dependent variability, making it difficult to maintain consistent precision and stability.

To overcome these limitations, automation technology integrating machine vision, image processing, and intelligent motion control has emerged as a key solution. By automatically detecting workpiece features and providing real-time position feedback, such systems achieve non-contact, high-speed, and high-accuracy alignment. Compared with manual methods, machine vision-based systems can reach sub-pixel positioning precision through algorithmic compensation and feature extraction, making them essential for micro- and nano-scale applications.

Despite these advances, existing alignment platforms still face major challenges. Single-CCD vision systems are limited by a narrow field of view and insufficient spatial coverage around the rotational center, which restrict their ability to detect and compensate for translational and rotational deviations in real time. In addition, conventional control methods struggle to ensure fast response and stability under nonlinear dynamics, external disturbances, and parameter uncertainties. These constraints hinder the precision and adaptability required in complex, high-density industrial environments.

To address these challenges, this study proposes an intelligent six-axis alignment platform featuring a dual-CCD vision configuration and an advanced nonlinear control strategy. The dual-CCD structure simultaneously captures feature points on both sides of the rotational center, enabling precise measurement and rapid correction of translational and rotational errors through geometric computation and image fusion. Meanwhile, the Manifold Deformation Design Scheme (MDDS) is adopted to enhance control robustness and dynamic response, achieving adaptive trajectory deformation and accurate motion control under uncertain conditions.

Overall, this research aims to overcome key bottlenecks in vision-based measurement, motion control, and system integration. The proposed dual-CCD six-axis platform achieves high precision, stability, and real-time performance, providing a reliable foundation for high-end industrial applications, and intelligent automated manufacturing.

### 1.1. State of the art review

To provide a comprehensive understanding of the current progress in precision alignment systems, this section is divided into three main parts: machine vision and image positioning, control methods for precision motion systems, and alignment platform design and applications. These areas collectively form the technological foundation for developing intelligent and high-precision alignment platforms.

#### 1.1.1. Machine vision and image positioning.

In industrial automation, machine vision image positioning is crucial. The image positioning methods are typically divided into the best-fit line method and the best-fit point method. For the best-fit line method, calculations are performed from the boundary points of features to determine the optimal line that approximates the feature. The best-fit point method calculates representative values for all pixels within a region. To enhance feature detection and reduce noise, edge detection and other image processing techniques are used to improve recognition accuracy. In image positioning research, Kang et al. [1] proposed an image processing technique for precise positioning of arbitrary images in holographic stereograms. Their method enhanced spatial alignment and image quality by optimizing placement through computational adjustments. The approach improved the accuracy of holographic reconstructions, ensuring better visual representation. Mohr et al. [2] explored positioning techniques using multiple images to enhance spatial understanding. Their study focused on extracting positional information through image analysis and geometric computations. From their experimental results, by using multi-view data, the approach validated accuracy in object localization and scene reconstruction. Shang-Hong Lai [3] proposed a robust image matching method that enhances feature correspondence even under occlusion and illumination changes. The approach utilized adaptive similarity measures and transformation models to improve reliability in object recognition and tracking. The experimental results validated its ability to maintain accuracy even in challenging environments. Devernay and Faugeras [4] addressed the preservation of straight lines in image processing and computer vision. They proposed a method ensuring that straight lines remain undistorted after camera calibration and rectification. Their approach improved accuracy in 3D reconstruction and image analysis, benefiting applications in machine vision and robotics. Kim et al. [5] developed a clustering-based template method for extracting text regions from images. Their approach enhanced robustness in complex environments by employing pattern recognition and clustering algorithms. In experimental results, the accurately detecting and extracting text regions across diverse image conditions were demonstrated its effectiveness. Reulke et al. [6] analyzed the spatial resolution of the ADS40 CCD line-scanner system and proposed methods for its improvement. They examined factors affecting resolution, including sensor geometry and image processing techniques. Their findings contributed to enhancing image quality and accuracy in photogrammetry and remote sensing applications. Lin et al. [7] investigated the use of visual servoing technology for high-precision mechanical positioning. Their approach integrated image processing techniques to enhance accuracy and reliability in positioning systems. The experimental results demonstrated its effectiveness in improving precision and stability, making it suitable for advanced mechanical applications. Sucar et al. [8] proposed iMAP, a real-time implicit mapping and positioning system using neural networks. It achieved accurate 3D scene reconstruction and camera tracking simultaneously, demonstrating efficiency and scalability in dynamic environments. Jing et al. [9] provided a comprehensive review of recent advances in image edge detection. It covered traditional methods, deep learning-based approaches, and hybrid techniques, analyzing their strengths, limitations, and applications. The authors discussed benchmark datasets, evaluation metrics, and future research directions, highlighting emerging trends in edge detection technology. Liu et al. [10] introduced PETR a position embedding transformation method for multi-view 3D object detection. PETR enhanced image features by encoding 3D positional information, enabling more accurate depth-aware object localization. Their results had significantly influenced image-based rendering by enabling high-quality visual realism and efficient scene representation. Wang et al. [11] proposed a fast and precise autofocus method based on a linear array CCD for optical metrology and industrial inspection. The method enhanced focus accuracy by optimizing image contrast evaluation and employing an efficient search algorithm. The approach was particularly beneficial for high-precision imaging applications in manufacturing and quality control.

#### 1.1.2. Control methods for precision motion systems.

When image positioning on the platform is completed, whether for the XXY platform or the XYZ axes, each movement must smoothly and accurately reach the target position. To achieve this goal, an accurate control method is required. In the study of control methods, Mizumoto [12] explored the realization of PID (Proportional-Integral-Derivative) control using fuzzy control methods, aiming to enhance system performance and robustness. The study presented a fuzzy logic-based approach to implement PID controllers, improving adaptability to nonlinear and uncertain systems. In experimental results, by adjusting control parameters dynamically, the method achieved better stability and response compared to conventional PID controllers. Uzmay et al. [13] explored robust and adaptive control techniques for cooperative manipulation to improve precision and stability by experimental methods. The study introduced control strategies that address uncertainties, external disturbances, and dynamic interactions among multiple manipulators. By merging adaptive and robust techniques, the system enhanced coordination, force regulation, and trajectory tracking, resulting in improved efficiency for complex manipulation tasks. Yao and Jiang [14] discussed advanced motion control, moving beyond classical PID to nonlinear adaptive robust control (ARC). They highlighted the limitations of PID controllers and introduced ARC to improve precision and stability. ARC addressed uncertainties and disturbances in dynamic systems by integrating adaptive learning with robust control. The experimental results demonstrated improved performance, highlighting the suitability of this approach for high-precision applications. Liu and Liu [15] proposed a MDDS (Manifold Deformation Design Scheme) for controlling nonlinear systems. By modifying system dynamics through manifold deformation, this approach efficiently managed uncertainties and disturbances in nonlinear control applications. The simulation and experimental results verified the system’s enhanced tracking accuracy and robustness. Na et al. [16] proposed a robust adaptive finite-time parameter estimation and control method for robotic systems. The approach ensured quick convergence and accurate parameter estimation by integrating finite-time control with adaptive estimation techniques. These results by simulation and experiment validated its effectiveness in enhancing robotic performance. Grüne and Pannek [17] explored nonlinear model predictive control (NMPC), focusing on its theoretical foundations and practical applications. The paper discussed NMPC’s ability to handle system constraints, uncertainties, and nonlinear dynamics by optimizing control inputs over a prediction horizon. Key topics included stability analysis, computational efficiency, and real-time implementation. Al-Shabi et al. [18] investigated robust nonlinear control and state estimation for a PRRR (Prismatic-Revolute-Revolute-Revolute) robot system. Their research presented advanced control techniques to enhance stability, accuracy, and disturbance rejection in highly nonlinear robotic systems. Both simulation and experimental results confirmed the effectiveness of the proposed control strategy, demonstrating its suitability for high-precision robotic applications. Hu et al. [19] introduced a robust adaptive fixed-time sliding-mode control strategy designed for uncertain robotic systems with input saturation. This approach significantly enhanced disturbance rejection and uncertainty compensation by integrating adaptive control with sliding-mode techniques, ensuring precise control throughout. The experimental results confirmed its effectiveness in improving system performance and robustness. Fu et al. [20] proposed a robust adaptive time-varying region tracking control method for multi-robot systems. The approach combined robust and adaptive control techniques to ensure accurate tracking and stability in time-varying regions. The simulation results confirmed the method’s capability to manage disturbances and ensure effective coordination in dynamic environments. Li et al. [21] reviewed recent advancements in flexible robotic manipulator systems, emphasizing dynamics modeling methods. The study explored various techniques, including finite element methods and machine learning-based approaches, highlighting their role in enhancing control precision and performance. Accurate dynamic modeling was crucial for applications in industrial automation, medical robotics, and space exploration.

#### 1.1.3. Alignment platform design and applications.

Alignment platforms are essential in industrial manufacturing, with XYZ platforms primarily used for long-distance positioning and XXY micro-positioning platforms commonly applied in predetermined control systems. Enhancing the precision of alignment platforms has become a key research focus. In the study of alignment platforms, Kwon and Lee [22] presented a microscopic motion control method for a parallel visual alignment stage. The system utilized high-precision control algorithms to achieve fine motion for visual alignment, improving accuracy and stability. In their experiment, these results validated its effectiveness in enhancing alignment precision for high-precision applications. Chih-Chin Wen [23] presented a machine vision integration method using an ultra-low alignment stage for precise positioning. The system enhanced accuracy and reliability through the use of advanced image processing and alignment algorithms. Their experimental results confirmed its effectiveness in high-precision manufacturing and optical inspection applications. Tsau et al. [24] introduced a multiple alignment stage for an automatic precision alignment system, aimed at enhancing both accuracy and efficiency in industrial applications. The system incorporated advanced motion control techniques, real-time feedback mechanisms, and high-precision positioning methods to achieve optimal alignment performance. Hau-Wei Lee [25] proposed an optical alignment system utilizing a parallel XXY stage and four CCDs for micro-pattern alignment. The system enhanced precision through multi-camera image processing and coordinated stage control. In their experiment, these results demonstrated significant improvements in alignment accuracy. Lin et al. [26] developed an optical alignment system using an XXY stage integrated with dual CCDs to enhance positioning accuracy. The system incorporated real-time image processing and precise motion control, improving alignment efficiency and stability. By utilizing dual CCDs, it achieved greater precision in detecting and correcting alignment errors. The experimental results validated its effectiveness in applications demanding high-precision alignment. Chen et al. [27] developed an image-based control system for an XXY positioning platform, enhancing precision through machine vision and advanced control algorithms. The system processed real-time image feedback to improve positioning accuracy, reducing errors in high-precision applications, from their experimental results. Kim et al. [28] developed a low-cost six-axis alignment instrument designed for flexible 2D and 3D ultrasonic probes. The system improved alignment accuracy and flexibility, making it suitable for various medical imaging and industrial applications. By combining cost-effective design with high precision, the instrument enhanced the performance of ultrasonic probes while maintaining affordability. Shaotran et al. [29] introduced “Aligned,” a platform-based process for alignment, designed to improve precision and efficiency in various applications. In their experiment, the platform integrated advanced alignment techniques and process optimization strategies, offering a scalable solution for manufacturing and automation. Huang and Chan [30] investigated enhancing the positioning accuracy of an XXY planar platform through computer vision and reinforcement learning. Their approach integrated reinforcement learning algorithms for real-time optimization, enabling precise adjustments to improve platform positioning. The effectiveness of the system in enhancing performance for industrial automation and precision control applications was confirmed by experimental results.

### 1.2. Research motivation and objectives

Despite continuous advancements in optical measurement and automation technologies, conventional alignment platforms still face significant challenges in achieving high-precision, real-time compensation for both translational and rotational deviations. In particular, single-CCD vision systems are limited by a restricted field of view and insufficient spatial coverage around the rotational center, which constrains their ability to accurately detect and correct workpiece attitude deviations. These limitations not only reduce positioning accuracy but also restrict operational flexibility in high-density production environments. Moreover, traditional control methods often struggle to maintain fast response, stability, and high precision simultaneously when dealing with nonlinear system dynamics, external disturbances, and parameter uncertainties, limiting their applicability in complex industrial scenarios. These challenges underscore the urgent need to develop advanced control strategies and optimize platform design for high-precision alignment applications.

To address these issues, the main objective of this study is to develop a high-precision, image-based positioning and control system for an alignment platform equipped with dual CCDs provided by the manufacturer, aiming to enhance the system’s overall accuracy and stability. The platform adopts a dual-CCD vision configuration, which captures key feature points simultaneously from both sides of the rotational center, enabling precise measurement and rapid correction of translational and rotational deviations. By detecting and linking key features on the workpiece surface, such as boundary edges, extension lines or fiducial markers, the system can extract pose variation information to determine both translational and rotational errors. Specifically, for rotational deviation detection, when feature points are located on opposite sides of the rotation center, the rotation angle can be directly and accurately determined through geometric relationships, allowing for fast and precise attitude correction. Compared with single-CCD systems, the dual-CCD configuration effectively expands the observation range, overcomes field-of-view limitations, and mitigates installation constraints, thereby improving real-time measurement accuracy and reliability.

In terms of control strategy, the alignment platform employs the Manifold Deformation Design Scheme (MDDS) [15], a nonlinear control method capable of dynamically adjusting motion trajectories based on system states while maintaining high robustness against system uncertainties and external disturbances. To evaluate the effectiveness of MDDS on this precision alignment platform, comparative experiments with conventional PID control were conducted. By analyzing the experimental results of both control approaches, the study verified the practical performance of MDDS in enhancing the platform’s control accuracy and stability.

Furthermore, to improve operational convenience and real-time monitoring, a user-friendly Human–Machine Interface (HMI) was developed to integrate the vision recognition and motion control modules. The HMI provides real-time visualization of system states, trajectory data, and alignment progress, enabling operators to adjust control parameters and optimize performance immediately, thereby enhancing operational flexibility and industrial applicability.

Overall, the motivation of this study is to address the technical bottlenecks of high-precision alignment platforms in visual measurement, control methodology, and system integration. The proposed dual-CCD six-axis alignment platform combines high accuracy, stability, and real-time responsiveness, providing a reliable technological solution for high-end industrial applications such as semiconductor manufacturing, optical component processing, and intelligent automated production.

## 2. Description of the physical problem

**Fig 1:**
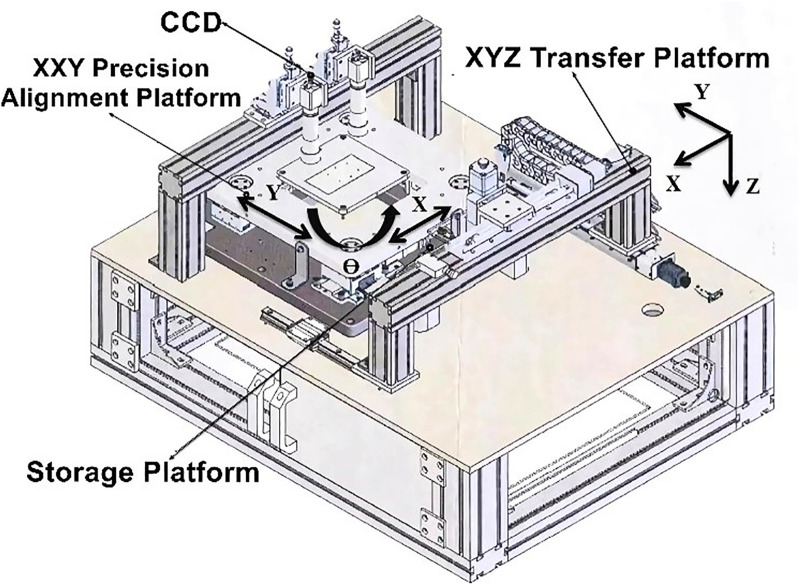
Schematic diagram of the six-Axis precision alignment platform structure.

To meet the partner company’s requirements for high-repeatability and high-precision alignment operations in automated production lines, this study designs and develops a six-axis precision alignment platform equipped with an image-based positioning and control system. This system replaces traditional manual alignment methods, improving production efficiency and positioning accuracy. The six-axis precision alignment platform consists of an XYZ platform and an XXY three-axis micro-displacement servo transfer platform, enabling multi-axis high-precision motion control. Additionally, the platform is equipped with dual CCD image acquisition devices, mounted on the system to capture real-time images of target objects. Using advanced image processing algorithms, the system achieves precise recognition and positioning. A schematic diagram of the platform structure is shown in Fig 1.

## 3. Methodology for the precision alignment platform

The XYZ platform of the precision alignment platform is driven by three mutually perpendicular actuators, consisting mainly of motors, a base, and ball screw guides, enabling three-dimensional movement. The XXY platform is driven by two X-axis actuators (X1, X2) and one Y-axis actuator (Y), with a structure that includes motors, a worktable, a base, and a follower module. The follower module comprises couplings, travel limit blocks, sensors, and cross-roller bearings. Between the base and the worktable, four flat surfaces are fitted with a MINI Stage XYθ-axis slide table assembly and cross-roller bearings. The platform’s translational and rotational movements are achieved through three sets of ball screw guides connected to the motors. These methods employed in the image positioning and control system of the six-axis precision alignment platform are detailed in the following sections.

### 3.1. Kinematics of the XXY alignment platform

Based on inverse kinematics, the variation in the center coordinates and rotation angle of the XXY platform is used to compute the coordinate changes of the MINI Stage through inverse kinematics calculations. This process determines the required rotation of each actuator [31–32].

**Fig 2:**
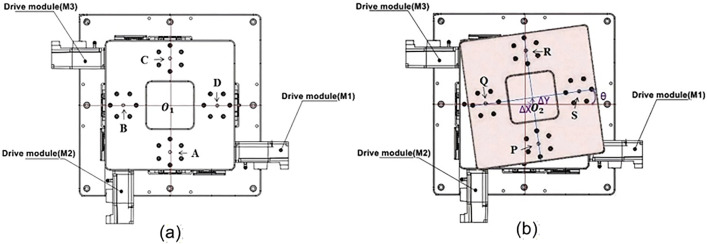
Schematic diagram of XXY platform. **(a)** Platform at Rest. **(b)** Platform in Rotation.


Assuming the world coordinate system is located at the center of the platform $O_{\text{1}}$, when the XXY platform is at rest, (A, B, C, D) = (P, Q, R, S), and $O_{\text{1}\;}$ = $O_{\text{2}}$. The platform’s center coordinates are $\left(x_{\text{1}},y_{\text{1}}\right)$ and the rotation angle is $\;\theta_{\text{1}}$, as shown in Fig 2(a). After the platform moves, the coordinates of $O_{\text{2}}$ are $\left(x_{\text{2}},y_{\text{2}}\right)$ and the rotation angle is $\theta_{\text{2}}$, as shown in Fig 2(b).

$$\vec{O_{1}O_{2}}=[\begin{array}{c} x_{\text{2}}-x_{\text{1}}\\ y_{\text{2}}-y_{\text{1}}\\ \theta_{\text{2}}-\theta_{\text{1}} \end{array}]=[\begin{array}{c} \Delta x\\ \Delta y\\ \Delta \theta \end{array}]$$ (1)

When at rest, the distances from the MINI stage center points (A, B, C, D) to $O_{\text{1}}$ are all equal to h.


$$\vec{O_{\text{1}}A}=\left(x_{A},y_{A}\right)=(\text{0},-h),\;\vec{O_{\text{1}}B}=\left(x_{B},y_{B}\right)=(-h,\text{0})$$

$$\vec{O_{\text{1}}C}=\left(x_{C},y_{C}\right)=(\text{0},h),\;\vec{O_{\text{1}}D}=(x_{D},y_{D})=(h,\text{0})\;$$ (2)

The center points of the MINI stage after movement are (P, Q, R, S), where the relationship between the coordinates before and after the movement is as follows:


$$\;\vec{O_{\text{2}}P}=R(\Delta \theta )\vec{O_{\text{1}}A}$$

$$\vec{O_{\text{2}}Q}=R(\Delta \theta )\vec{O_{\text{1}}B}$$

$$\vec{O_{\text{2}}R}=R(\Delta \theta )\vec{O_{\text{1}}C}$$

$$\vec{O_{\text{2}}S}=R(\Delta \theta )\vec{O_{\text{1}}D}$$ (3)

The two-dimensional rotation matrix is represented as:


$$R(\Delta \theta )=[\begin{array}{cc} cos\Delta \theta & -sin\Delta \theta \\ sin\Delta \theta & cos\Delta \theta \end{array}]$$ (4)

The relationship between the points (P, Q, R, S) can be expressed using spatial vectors as follows:


$$\vec{O_{\text{1}}\;\text{P}}=\vec{O_{\text{1}}O_{\text{2}}\;}+\vec{O_{\text{2}}P}=\vec{O_{\text{1}}O_{\text{2}}\;}+R(\Delta \theta )\vec{O_{\text{1}}A}$$

$$\vec{O_{\text{1}}\;\text{Q}}=\vec{O_{\text{1}}O_{\text{2}}\;}+\vec{O_{\text{2}}Q}=\vec{O_{\text{1}}O_{\text{2}}\;}+R(\Delta \theta )\vec{O_{\text{1}}B}$$

$$\vec{O_{\text{1}}\;\text{R}}=\vec{O_{\text{1}}O_{\text{2}}\;}+\vec{O_{\text{2}}R}=\vec{{O_{\text{1}}O}_{\text{2}}\;}+R(\Delta \theta )\vec{O_{\text{1}}C}$$

$$\vec{O_{\text{1}}\;\text{S}}\;=\vec{O_{\text{1}}O_{\text{2}}\;}+\vec{O_{\text{2}}S}=\vec{O_{\text{1}}O_{\text{2}}\;}+R(\Delta \theta )\vec{O_{\text{1}}D}$$ (5)

Substitute equations (2), (3), and (4) into equation (5) to obtain equation (6).


$$\left[\begin{array}{c} x_{P}\\ y_{P} \end{array}]=[\begin{array}{c} \Delta x\\ \Delta y \end{array}]+[\begin{array}{cc} cos\Delta \theta & -sin\Delta \theta \\ sin\Delta \theta & cos\Delta \theta \end{array}][\begin{array}{c} \text{0}\\ -h \end{array}]=[\begin{array}{c} \Delta x+hsin\Delta \theta \\ \Delta y-hcos\Delta \theta \end{array}\right]$$

$$\left[\begin{array}{c} x_{Q}\\ y_{Q} \end{array}]=[\begin{array}{c} \Delta x\\ \Delta y \end{array}]+[\begin{array}{cc} cos\Delta \theta & -sin\Delta \theta \\ sin\Delta \theta & cos\Delta \theta \end{array}][\begin{array}{c} -h\\ \text{0} \end{array}]=[\begin{array}{c} \Delta x-hcos\Delta \theta \\ \Delta y-hsin\Delta \theta \end{array}\right]$$

$$\left[\begin{array}{c} x_{R}\\ y_{R} \end{array}]=[\begin{array}{c} \Delta x\\ \Delta y \end{array}]+[\begin{array}{cc} cos\Delta \theta & -sin\Delta \theta \\ sin\Delta \theta & cos\Delta \theta \end{array}][\begin{array}{c} \text{0}\\ h \end{array}]=[\begin{array}{c} \Delta x-hsin\Delta \theta \\ \Delta y+hcos\Delta \theta \end{array}\right]$$

$$\left[\begin{array}{c} x_{S}\\ y_{S} \end{array}]=[\begin{array}{c} \Delta x\\ \Delta y \end{array}]+[\begin{array}{cc} cos\Delta \theta & -sin\Delta \theta \\ sin\Delta \theta & cos\Delta \theta \end{array}][\begin{array}{c} h\\ \text{0} \end{array}]=[\begin{array}{c} \Delta x+hcos\Delta \theta \\ \Delta y+hsin\Delta \theta \end{array}\right]$$ (6)

The changes in each axis [X1, Y, X2]^T^ are expressed as:


$$\left[\begin{array}{c} X\text{1}\\ Y\\ X\text{2} \end{array}]=[\begin{array}{c} -\Delta x\\ \Delta y\\ \Delta x \end{array}]=[\begin{array}{c} -(x_{P}-x_{A})\\ y_{Q}-y_{B}\\ x_{R}-x_{C} \end{array}]=[\begin{array}{c} -\Delta x-hsin\Delta \theta \\ \Delta y-hsin\Delta \theta \\ \Delta x-hsin\Delta \theta \end{array}\right]$$ (7)

Forward kinematics involves calculating the displacement [X1, Y, X2]^T^ of the MINI stage based on the known rotation of each axis. By deriving the XXY platform’s center coordinates and rotation angle through forward kinematics, the exact position and orientation of the XXY platform can be determined [33–34].

Similarly, assuming the world coordinate system is located at the platform center $O_{\text{1}}$, the positions of the XXY platform in both stationary and rotated states are shown in Fig 2. The XXY platform is driven by two X-axis actuators (X1, X2) and one Y-axis actuator (Y) to achieve movement. When the platform undergoes pure X-direction translation, the M1 and M3 drive modules generate equal but opposite displacements, resulting in platform movement along the X-axis. For pure Y-direction translation, only the M2 drive module needs to be activated to move the platform along the Y-axis.


$$\left[\begin{array}{c} X\text{1}\\ Y\\ X\text{2} \end{array}]=[\begin{array}{c} -\Delta x\\ \Delta y\\ \Delta x \end{array}\right]$$ (8)

**Fig 3:**
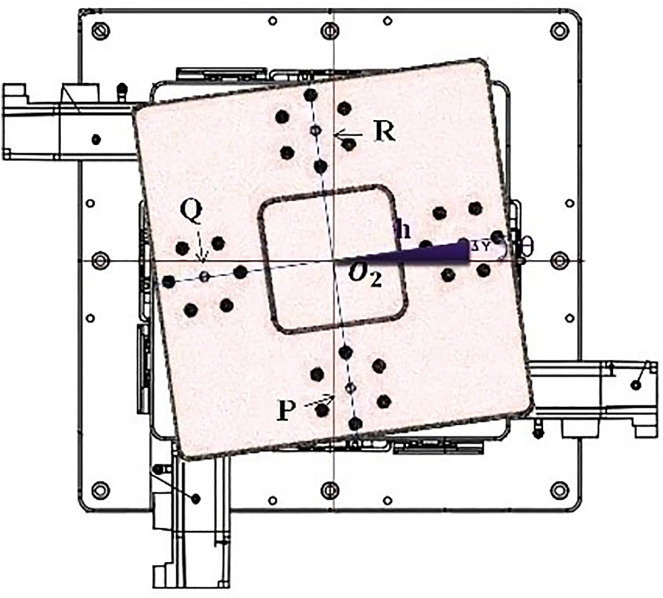
XXY rotation coordinate diagram.


The distance (h) from the MINI stage to the platform center is equal in all directions. When the platform undergoes pure rotation, all three axes will experience the same displacement (Fig 3).

$$\left[\begin{array}{c} X\text{1}\\ Y\\ X\text{2} \end{array}]=[\begin{array}{c} hsin\Delta \theta \\ hsin\Delta \theta \\ hsin\Delta \theta \end{array}\right]$$ (9)

From Equations (1), (2), (8), and (9), we obtain $[\Delta x,\Delta y,\Delta \theta {]}^{T}$


$$\left[\begin{array}{c} \Delta x\\ \Delta y\\ \Delta \theta \end{array}]=[\begin{array}{c} x_{\text{2}}-x_{\text{1}}\mathrm{\backslash vspace}1mm\\ y_{\text{2}}-y_{\text{1}}\mathrm{\backslash vspace}1mm\\ \left(\theta_{\text{2}}-\theta_{\text{1}}\right) \end{array}]=[\begin{array}{c} \frac{-X\text{1}+X\text{2}}{\text{2}}\mathrm{\backslash vspace}1mm\\ \frac{-(X\text{1}+X\text{2})}{\text{2}}+Y\mathrm{\backslash vspace}1mm\\ Sin^{-\text{1}}(\frac{-(X\text{1}+X\text{2})}{\text{2}h}) \end{array}\right]$$ (10)

### 3.2. System model of the XXY platform

The XXY platform consists of three actuators. Two of them are aligned horizontally with the X-axis of the coordinate system, facing the positive and negative directions of the X-axis, respectively. The third actuator is positioned vertically relative to the X-axis and faces the positive direction of the Y-axis. These three actuators work together to control the X and Y translations and the rotation angle θ of the worktable.

In the kinematic model, the velocity of the XXY platform can be obtained by mapping the actuator velocities through the Jacobian matrix, as expressed in Equation (11):


$$\dot{P}=J\dot{q\;}$$ (11)

Where $\dot{P}$ denotes the velocity and angular velocity vector of the platform center, defined as


$$\dot{P}=[\begin{array}{ccc} \dot{x} & \dot{y} & \dot{\theta } \end{array}{]}^{T}$$ (12)

J is the Jacobian matrix of the XXY platform, expressed as


$$J=[\begin{array}{ccc} -\frac{\text{1}}{\text{2}} & \text{0} & \frac{\text{1}}{\text{2}}\mathrm{\backslash vspace}1.5mm\\ -\frac{\text{1}}{\text{2}} & \text{1} & -\frac{\text{1}}{\text{2}}\mathrm{\backslash vspace}1.5mm\\ -\frac{\text{1}}{\text{2}hcos(\Delta \theta )} & \text{0} & -\frac{\text{1}}{\text{2}hcos(\Delta \theta )} \end{array}]$$ (13)

$\dot{q\;}$ represents the velocity vector of the three linear actuators, given by


$$\dot{q}={\left[{\dot{X}}_{\text{1}},\;\;\dot{Y,}{\dot{X}}_{\text{2}}\;\right]}^{T}$$ (14)

Here, ${\dot{X}}_{\text{1}}$ and ${\dot{X}}_{\text{2}}\;$ are the velocities of the two horizontal actuators aligned with the X-axis, and $\dot{\;Y\;}$ is the velocity of the vertical actuator aligned with the Y-axis. These three actuators jointly generate translational motion in the X–Y plane and rotational motion $\dot{\theta \;}$ of the worktable around its center.

### 3.3. Image recognition method

This study develops a dual-camera image recognition method for scene identification and spatial analysis to obtain the pose information of target objects. Based on the recognition results, the control module coordinates the movement of each actuator axis, allowing the platform to reach the target position with high speed and precision. To enhance the accuracy and stability of recognition and positioning, the proposed image recognition approach combines two core techniques, image processing and image positioning, ensuring high-quality visual data for subsequent spatial localization and alignment control.

#### 3.3.1. Image processing technology.

In the image processing stage, the system processes the raw images captured by the dual cameras through a series of operations, as outlined below:

(1) **Grayscale and binarization**

When converting a color image to a grayscale image, the grayscale value is calculated by a weighted combination of the red, green, and blue components as follows:


Z=0.30R+0.59G+0.11B (15)

where  Z   represents the grayscale intensity, and R, G, and B denote the brightness levels of the red, green, and blue channels, respectively. This conversion preserves the brightness characteristics of the original image while mapping colors into corresponding grayscale levels.

After grayscale conversion, a binarization process is applied, setting each pixel value to either 0 or 255 to create a distinct black-and-white effect. This process significantly reduces the image’s data volume and enhances the visibility of object contours. Grayscale and binarization processing play an essential role in digital image processing, providing a clean and high-contrast foundation for subsequent edge detection and feature extraction operations [35–36].

(2) **Median filtering**

Median filtering is a typical nonlinear filtering technique. Its principle is to replace the gray value of a pixel with the median of the gray values of its surrounding neighborhood pixels. The operation can be expressed as:


E(x,y)=median{s(i,j)|(i,j)∈W(x,y)} (16)

where s(i,j) represents the gray value of the pixel at position (i,j), W(x,y) denotes the neighborhood window centered at (x,y), and E(x,y) is the resulting filtered pixel value.

This method effectively suppresses noise while preserving image edges and fine structural details. It is particularly suitable for removing speckle noise and salt-and-pepper noise, as it does not rely on extreme values in the neighborhood. Compared with linear filtering methods such as mean or least mean square filtering, median filtering provides better smoothing performance with less blurring, making it one of the most widely used preprocessing techniques for digital image denoising [37].

(3) **Dilation and erosion**

Dilation and erosion are fundamental morphological operations extensively used for analyzing and processing the structural characteristics of binary images. The core concept involves performing set operations between the image and a defined structuring element to achieve expansion or contraction of image regions.

For a given image set  A and structuring element B, the dilation operation is defined as:


$$A⨁B=\left\{l|{\left(\hat{B}\right)}_{l}\cap A\neq \text{0}\right\}$$ (17)

where $\;\hat{B}\;$ represents the reflection of B about the origin, and ${\left(\hat{B}\right)}_{l}\;$ denotes the translation of $\hat{B}$ by vector l. When at least one pixel within the neighborhood corresponding to the structuring element equals 255, the output pixel is set to 255. Dilation expands the boundaries of bright regions in an image and is often used for filling small gaps or connecting adjacent objects.

Conversely, the erosion operation is defined as:


$$A⊖B=\left\{l|B_{l}\subseteq A\right\}$$ (18)

That is, when the structuring element B completely fits within the region A at position l, the output pixel is assigned a value of 255. Erosion effectively shrinks object boundaries and removes isolated noise or unwanted small structures.

Based on the above explanation, dilation and erosion are complementary operations commonly used for morphological optimization, such as boundary smoothing, hole filling, and noise removal. They also serve as the foundation for higher-level morphological processes like opening and closing operations [38].

(4) **Edge detection**

A descriptive Sobel edge detection algorithm is applied to extract contour features from the preprocessed images. The Sobel operator calculates image intensity gradients in horizontal and vertical directions using 3 × 3 convolution kernels, emphasizing regions with significant intensity variation. The gradient magnitude and direction are computed as:$G_{x}=[\begin{array}{ccc} -\text{1} & \text{0} & +\text{1}\\ -\text{2} & \text{0} & +\text{2}\\ -\text{1} & \text{0} & +\text{1} \end{array}]*I,\;G_{y}=[\begin{array}{ccc} +\text{1} & +\text{2} & +\text{1}\\ \text{0} & \text{0} & \text{0}\\ -\text{1} & -\text{2} & -\text{1} \end{array}]*I$


$$G=\sqrt{G_{x}^{\text{2}}+G_{y}^{\text{2}}},\;\theta ={tan}^{-\text{1}}\left(\frac{G_{y}}{G_{x}}\right)$$ (19)

where ${\;G}_{x}$ and ${\;G}_{x}$ denote the horizontal and vertical gradient components, respectively, I represents the input image.

A threshold is then applied to suppress weak gradients and retain only significant edges, thereby enhancing contour clarity and providing reliable geometric information for subsequent analysis [39].

(5) **Template matching**

Template matching is a technique used to locate regions within a target image that most closely resemble a given template image. The fundamental principle involves sliding the template image across the source image pixel by pixel and calculating a similarity measure at each position to evaluate the degree of correspondence between the template and the local region of the image. The similarity is typically computed using the Sum of Squared Differences method, as expressed in Equation (20):


$$R(x,y)=\sum {\mathrm{\backslash nolimits}}_{i=\text{0}}^{w-\text{1}}\sum {\mathrm{\backslash nolimits}}_{j=\text{0}}^{h-\text{1}}{\left[T(i,j)-I(x+i,y+i)\right]}^{\text{2}}$$ (20)

where R(x,y) represents the matching error value at position (x,y) in the result matrix, T(i,j) is the pixel value of the template image, and I(x+i,y+i) is the corresponding pixel value in the source image. A smaller matching error indicates a higher similarity, and the position with the minimum error corresponds to the best-matched region. By setting a threshold, irrelevant matches can be filtered out, ensuring accurate target localization. This method is computationally efficient, conceptually straightforward, and widely applied in object detection and visual positioning tasks [40].

(6) **Hough line detection**

Hough line detection is a widely used image processing technique for identifying and extracting linear features from binary images. The fundamental principle assumes that any edge pixel in the image may belong to a potential line. Through parameter space transformation, each line can be represented as a point in polar coordinates, where the corresponding line is perpendicular to the radius drawn from the origin to that point. The detection process can thus be interpreted as searching, for each pixel, all possible lines passing through it and accumulating votes in the parameter space. When multiple pixels vote for the same parameter values, a line is detected in the original image.

In practical applications, even if the line is slightly curved or discontinuous, the Hough Transform can still detect multiple short, closely aligned segments that approximate the original line. In the Cartesian coordinate system, a line can be represented as y=kx+b. However, when the slope k approaches infinity, this form becomes unsuitable. Therefore, the line is typically represented in polar coordinates as:


ρ=xcosθ+ysinθ (21)

where ρ denotes the perpendicular distance from the origin to the line, and θ represents the angle between this perpendicular line and the x-axis. By mapping all nonzero pixels from the image space into the (ρ, θ) parameter space, points that accumulate into prominent peaks correspond to detected lines in the image. Owing to its robustness against noise and discontinuous edges, the Hough Line Detection method is widely applied in boundary extraction and geometric feature analysis [41–42].

(7) **Overall image processing flow**

**Fig 4:**
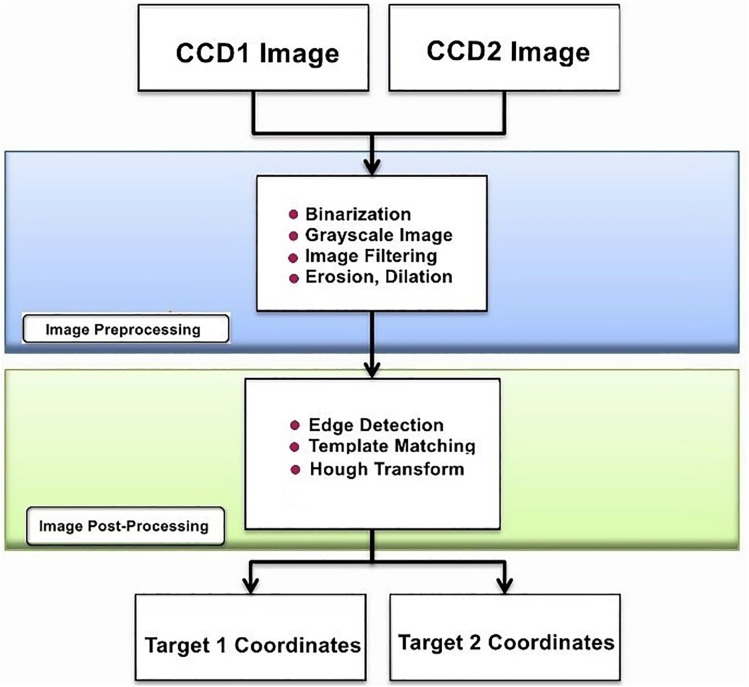
Flowchart of image processing.

This study employs dual CCD cameras for image acquisition, capturing color images of Target 1 and Target 2. The image processing workflow, as illustrated in Fig 4, aims to extract geometric features for precise coordinate calculations and application analysis. To enhance accuracy and reliability, various optimization techniques are implemented throughout the process to reduce noise, enhance target features, and improve detection precision.

During the preprocessing stage, images captured by CCD1 and CCD2 are first converted to grayscale to reduce data complexity while retaining brightness information for efficient processing. The images are then binarized to enhance contrast, making target features more distinct. To mitigate noise caused by environmental factors, median filtering is applied, effectively reducing random noise interference. Additionally, dilation and erosion operations are used to refine image quality—dilation fills small gaps to maintain complete target shapes, while erosion removes scattered noise, improving clarity and accuracy.

In the post-processing stage, edge detection is performed using the Sobel operator, which highlights gradient changes and extracts precise contour information. Once edges are identified, template matching is used to compare detected objects with predefined templates, ensuring accurate target positioning. Furthermore, the Hough Transform is applied for line detection, effectively identifying geometric structures and refining edge localization. By utilizing the Hough Transform, the system accurately extracts equations of the target’s edge lines, ensuring computational stability and precision.

These techniques collectively optimize the overall image processing workflow, significantly improving the accuracy and reliability of target detection and positioning.

#### 3.3.2. Image positioning technology.

**Fig 5:**
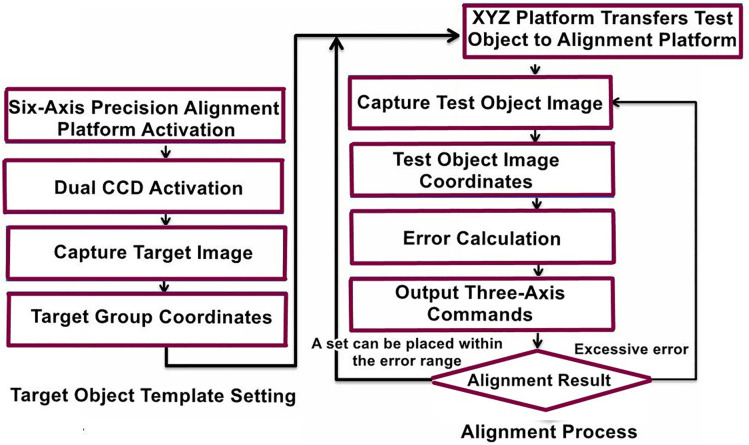
Image localization process flowchart.

For image positioning, dual CCD cameras are used for image acquisition. First, the target template coordinates must be set. After powering on the precision alignment platform, CCD1 and CCD2 respectively capture images of the target template. Through the image processing workflow, the acquired images undergo a series of analytical processes to obtain the coordinates of the target template’s markers: $T_{\text{1}}$ = ($T_{\text{1}x}$, $T_{\text{1}y})$ and $T_{\text{2}}$=($T_{\text{2}x}$, $T_{\text{2}y}$). Next, the object to be positioned is placed on the loading platform and transferred to the XXY alignment platform via the XYZ platform. The dual CCD cameras then capture images of the object to be positioned, and after image processing, the coordinates of its markers are obtained as $L_{\text{1}}$ =($L_{\text{1}x}$, $L_{\text{1}y}$) and $L_{\text{2}}$=($L_{\text{2}x}$, $L_{\text{2}y}$). Using the four sets of coordinates—$T_{\text{1}}$, $T_{\text{2}}$, $L_{\text{1}}$, and $L_{\text{2}}$—the command values for the three-axis actuators of the XXY platform are calculated. The alignment result is then evaluated. If the error is too large, the positioning process is repeated (capturing the image of the object, obtaining the marker coordinates, and recalculating the actuator movements). Otherwise, the next object is placed for positioning, and the process begins again. The positioning workflow is illustrated in Fig 5.

**Fig 6:**
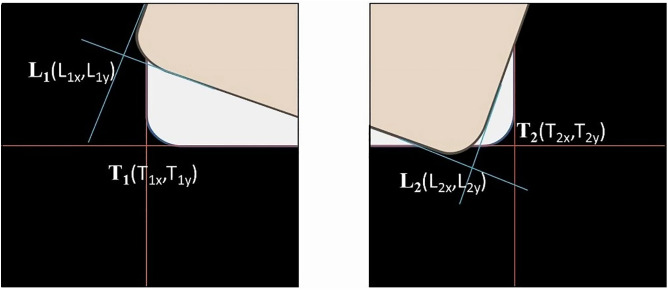
Schematic diagram of image positioning coordinates.


The target set coordinates $T_{\text{1}}$, $T_{\text{2}}$ and the object to be positioned set coordinates $L_{\text{1}}$, $L_{\text{2}}$, as shown in Fig 6, are used to obtain the center point coordinates of the vectors through equations (22) and (23).

$$C_{T}=\left(\frac{T_{\text{1}x}+T_{\text{2}x}}{\text{2}},\frac{T_{\text{1}y}+T_{\text{2}y}}{\text{2}}\right)$$ (22)

$$C_{L}=\left(\frac{L_{\text{1}x}+L_{\text{2}x}}{\text{2}},\frac{L_{\text{1}y}+L_{\text{2}y}}{\text{2}}\right)$$ (23)

By subtracting equations (22) and (23), the errors Δx and Δy can be obtained.


$$\Delta x=C_{Tx}-C_{Lx}=\frac{T_{\text{1}x}-L_{\text{1}x}+T_{\text{2}x}-L_{\text{2}x}}{\text{2}}$$ (24)

$$\Delta y=C_{Ty}-C_{Ly}=\frac{T_{\text{1}y}-L_{\text{1}y}+T_{\text{2}y}-L_{\text{2}y}}{\text{2}}$$ (25)

**Fig 7:**
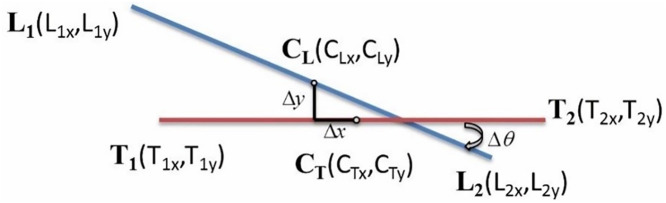
Rotation angle error between the target object and the object to be aligned.


Next, the rotation angle error Δθ is calculated as shown in Fig 7. The vectors between the target points $T_{\theta }$ and the object to be positioned points $L_{\theta }\;$ are measured with the X-axis of the coordinate system to find the angle for each, and then the difference between them is calculated to obtain the rotation angle error  Δθ.

$$\Delta \theta =T_{\theta }-L_{\theta }={tan}^{-\text{1}}\left(\frac{T_{\text{2}y}-T_{\text{1}y}}{T_{\text{2}x}-T_{\text{1}x}}\right)-{tan}^{-\text{1}}\left(\frac{L_{\text{2}y}-L_{\text{1}y}}{L_{\text{2}x}-L_{\text{1}x}}\right)$$ (26)

## 4. Construction of the precision alignment platform system

The precision alignment platform achieves multi-degree-of-freedom precise motion through a multi-axis motion control system. Combined with dual CCD cameras and image processing technology, it can accurately identify the target position and perform high-precision alignment. The construction of the entire experimental platform is described as follows:

### 4.1. System architecture

**Fig 8:**
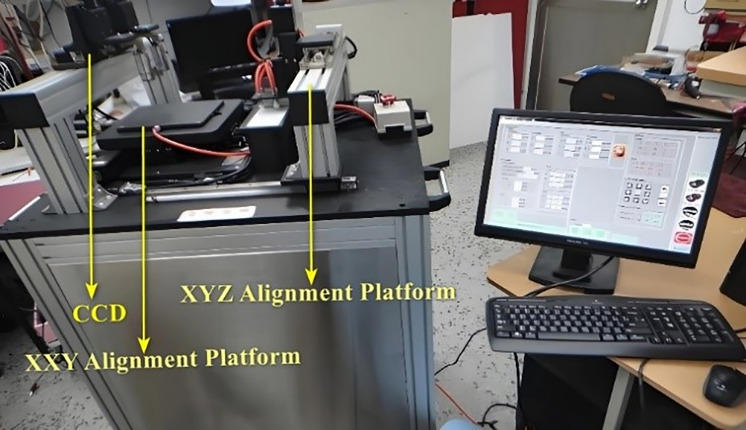
Construction of six-axis precision alignment platform.

The six-axis precision alignment platform in this study is provided by a collaborating manufacturer. The platform consists of a three-axis XXY alignment platform and a three-axis XYZ platform. For precise image alignment, a CCD camera with a 0.3x telecentric lens is installed above the XXY platform, as shown in the camera setup diagram in Fig 8. Each axis of the XXY alignment platform is driven by a 100W AC servo motor and equipped with a rotary encoder with 40,000 pulses per revolution to provide feedback on the motor’s position. The XYZ platform utilizes an air pressure system that generates internal pressure by compressing air, allowing it to pick up objects and move them to the XXY platform for alignment. The control system outputs control signals from a computer, which are amplified by an amplification circuit to regulate the switching of the air pressure system. The CCD serves as one of the feedback signals for the platform and provides the required coordinate information through image processing techniques.

**Fig 9:**
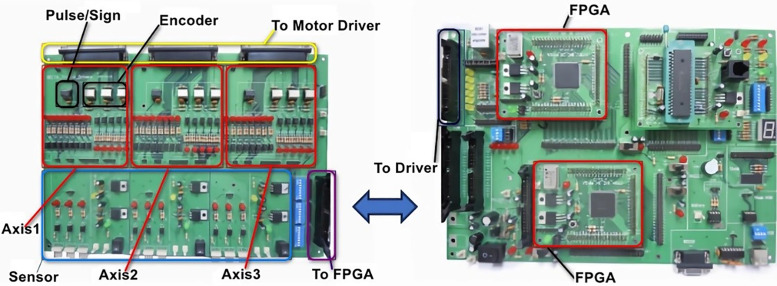
The connection diagram between the driver board and the motion control board.

For the motion of each axis of the XXY platform, the terminal signals from the servo motors of each axis are connected through the motor driver board and Field-Programmable Gate Array (FPGA) [43]. These signals, including pulse signals, direction signals, and encoder signals, are processed and then sent to the driver or the FPGA for computation (Fig 9). Due to FPGA’s high stability and parallel processing capabilities, FPGA is used as the core of the motion module, achieving multi-axis motion control. During the control process, because FPGA itself does not have arithmetic logic unit (ALU) functionality, a combination of FPGA and PC-based systems is used to construct a servo stepper control system.

**Fig 10:**
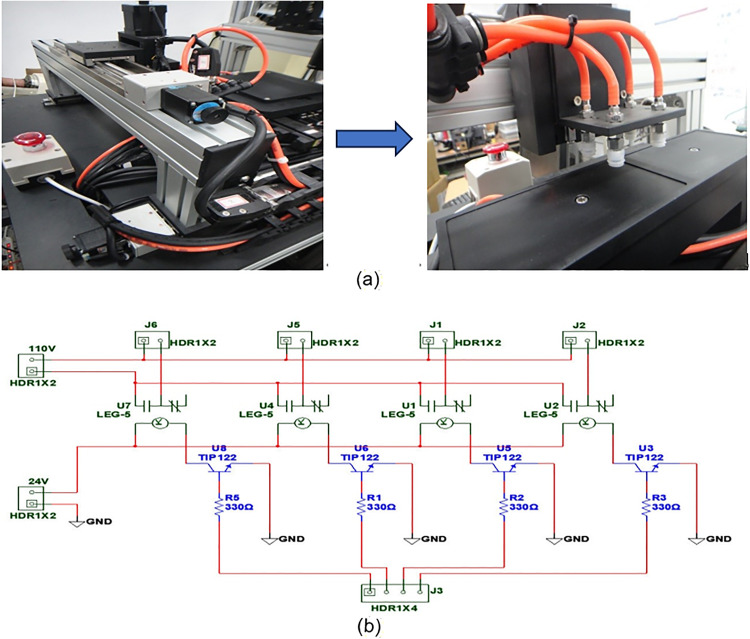
Diagram of the pneumatic system installed on the XYZ platform. **(a)** Object suction device of the XYZ alignment platform. **(b)** Pneumatic interface circuit design diagram.

The XYZ platform is equipped with an air pressure system, as shown in the installation schematic in Fig 10. This system utilizes the compressibility of air to regulate internal pressure, enabling the gripping and transportation of workpieces (Fig 10(a)). When a workpiece needs to be transferred to the XXY alignment platform for precise positioning, the system generates a vacuum to securely hold the workpiece with suction action, ensuring reliable and stable handling and positioning. In terms of control, the air pressure system is actuated by control signals sent from a computer. These signals are first amplified by a Darlington amplifier circuit to meet the current requirements for operating the air pressure switches and then delivered to the air pressure system for precise control of the switches (Fig 10(b)). This design ensures that the control signals can efficiently and stably drive the air pressure system, enabling automated and repeatable workpiece handling.

### 4.2. Human-Machine Interface

**Fig 11:**
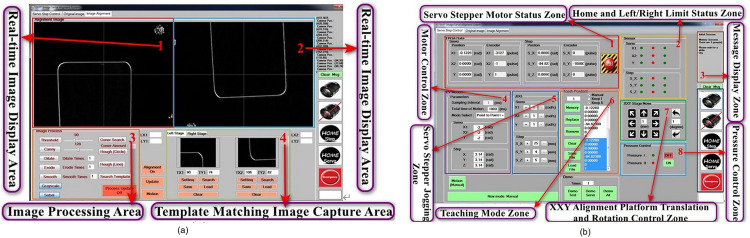
Human-Machine Interface for the Six-Axis Precision Alignment Platform. **(a)** Image Recognition Interface. **(b)** Multi-axis control motion interface.

The six-axis precision image positioning system is processed by a computer writing Visual Studio C++ for the human-machine interface. It uses image processing and the computer’s computational power to calculate motion paths and interpolate. The results are sent to the FPGA, which converts them into pulse signals to control motor movements. Based on the platform’s operational requirements, the human-machine interface design is divided into two major parts: image recognition and motion control. The image recognition interface, as shown in Fig 11(a), requires image processing technologies such as grayscale conversion, binarization, smoothing, and line detection, which are essential for image tracking and positioning. As for the platform’s single-axis or multi-axis movement, point-to-point motion control and trajectory tracking motion control interfaces are shown in Fig 11(b). The overall system, through the functional modules of the Human-Machine Interface (HMI), achieves high-precision image positioning and flexible motion control, improving the platform’s stability and accuracy.

### 4.3 System identification

The goal of System Identification is to obtain system parameters, understand the dynamic characteristics of the system, and perform optimal analysis and control. In this study, the time-domain identification method is used for open-loop identification of the motor system on the experimental platform to analyze the characteristics of the motors used. The motor speed control is considered as a first-order system, which contains one pole. The mathematical model of the input-output relationship can be represented as:


$$G(s)=\frac{Y(s)}{R(s)}=\frac{b}{s+a}=\frac{b}{a}\cdot \frac{\text{1}}{\frac{s}{a}+\text{1}}$$ (27)

**Fig 12:**
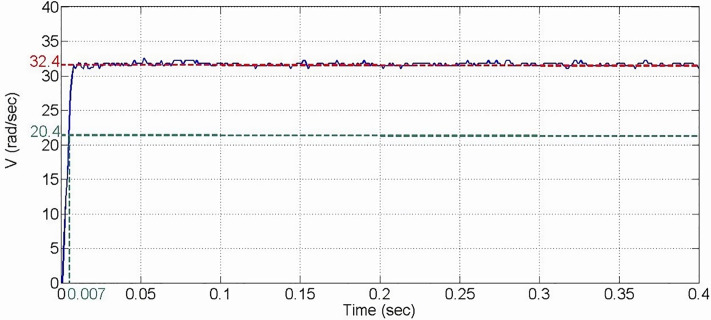
Open-loop identification response diagram of the motor system.

Where R(s) is the system input signal, measured in volts, and Y(s) is the motor output speed, measured in radians per second (rad/sec). By applying a step input command, the system’s step response can be observed, as shown in Fig 12. The system parameters can be identified by analyzing the following two indicators.

Steady-State Value: The system’s output quantity when it reaches a steady state.


$$G(\infty )=\frac{b}{a}$$ (28)

Time Constant (T): The time required to reach 63% of the steady-state value, T = 1/a.


$${\;C(t)|}_{t=\text{1}/a}={\;\text{1}-e^{-at}|}_{t=\text{1}/a}=\text{0}.\text{6}\text{3}$$ (29)

The step command used is as follows, with the unit in volts.


$$u\left(t\right)=\left\{\begin{array}{l} 0\\ 1 \end{array}\right)\begin{array}{l} ,\\ , \end{array}\begin{array}{l} t<0\;\text{sec}\\ t\geq 0\;\text{sec} \end{array}$$ (30)

The steady-state value of the system is given as follows.


$$G(\infty )=\frac{b}{a}=\text{32}.\text{4}\;\text{rad}/\text{sec}$$ (31)

The system’s time constant is 0.007 seconds. Based on equations (29) and (31), the system parameters are determined as a = 142 and b = 4600.8.

The relationship between the motor’s input signal and position can be represented using the identified parameters in the state-space equation, as shown in equation (32).


$$\left[\begin{array}{c} {ẋ}_{1}\\ {ẋ}_{2} \end{array}\right]=\left[\begin{array}{cc} 0 & 1\\ 0 & 142 \end{array}\right]\left[\begin{array}{c} x_{1}\\ x_{2} \end{array}\right]+\left[\begin{array}{c} 0\\ 4600.8 \end{array}\right]u$$ (32)

### 4.4. System control methods

**Fig 13:**
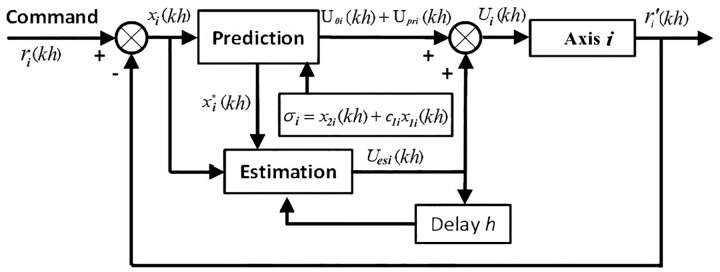
Block diagram of the MDDS control method for the i-th Axis.

The trajectory motion of each axis in the XXY platform of the six-axis precision alignment system is controlled using two approaches: MDDS [15] and PID control [12]. The MDDS method defines a sliding plane in the state space as a reference trajectory for system dynamics. A closed-loop feedback mechanism continuously compares the current state with this reference trajectory, computing and applying control signals to guide the system toward the sliding plane. Mechanical systems are often affected by disturbances such as gravity, friction, and inertia, leading to control errors. To address these problems, MDDS integrates predictive capabilities, estimating the system’s expected behavior at the next sampling point. If a deviation occurs between the actual and predicted states, real-time compensation is applied. This ensures the system remains stable on the sliding plane, achieving precise control while minimizing external disturbances’ impact on performance. As MDDS operates as a one-dimensional control method, each axis of the XXY platform can be individually treated as a single-axis manifold deformation control system, as illustrated in Fig 13.

The mathematical formula for the single-axis MDDS control method is as follows:


$$u(kh)=-\frac{f\left(x(kh)\right)}{b}-\frac{\sum_{n=\text{1}}^{n-\text{1}}c_{i}\left[x_{i}(kh)+h\cdot x_{i+\text{1}}(kh)\right]+x_{n}(kh)}{b\cdot h}+u_{es}(kh)$$ (33)

With


$$u_{es}(kh)=u_{es}(kh-h)-\frac{x_{n}(kh)-x_{n}^{*}(kh)}{\left(b\cdot h\right)}$$

$$x_{n}^{*}(kh+h)=-\sum_{i=\text{1}}^{n-\text{1}}c_{i}[x_{i}(kh)+h\cdot x_{i+\text{1}}(kh)]$$

In these expressions, u(kh) denotes the control input at the discrete sampling instant kh, and $x(kh)={\left[x_{\text{1}}(kh),x_{\text{2}}(kh),\ldots ,x_{n}(kh\right]}^{T}$ represents the system state vector. The parameter b corresponds to the system input gain, and $c_{i}$ are feedback coefficients for the respective state variables. The function f(x(kh)) describes the intrinsic nonlinear dynamics of the system; thus, the first term −f(x(kh))/b serves as a nonlinear compensation component that enhances the robustness and tracking accuracy of the control system. The second term implements a discrete linear combination of the system states and their forward differences, contributing to trajectory correction and stability. The term $u_{es}(kh$provides an additional error compensation mechanism, which is recursively updated based on the deviation between the current state $x_{n}(kh)$ and the predicted reference $x_{n}^{*}(kh)$. The predicted reference $x_{n}^{*}(kh+h)$ is calculated from a weighted combination of the previous states and their forward differences using the coefficients $c_{i}$. Overall, this control structure integrates nonlinear compensation, linear state feedback, and residual error correction, thereby enabling precise and robust trajectory tracking for nonlinear dynamic systems under discrete-time implementation.

In practical engineering applications, the most widely used control method is PID control. PID calculates the proportional (P), integral (I), and derivative (D) components based on the system error to generate the corresponding control signal, achieving precise regulation. The mathematical expression for single-axis PID control is as follows:


$$U(t)=K_{p}X(t)+K_{i}\int_{\text{0}}^{t}X(\tau )d\tau +K_{d}\frac{dX(t)}{dt}$$ (34)

**Fig 14:**
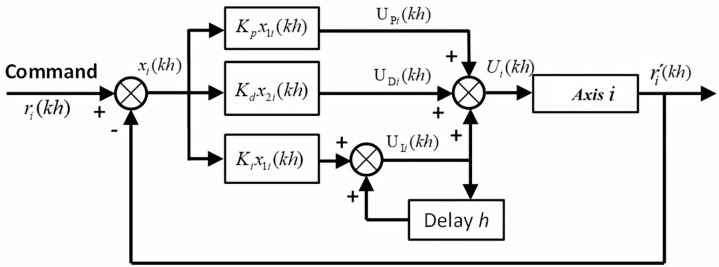
Block diagram of the PID control method for the i-th axis.


The control system used in this study is a digital control system, where time is not continuous. Therefore, Equation (34) is rewritten as Equation (35), where h is the sampling time of the digital system, and k is the sampling index, as shown in Fig 14.

$$U(kh)=U_{P}(kh)+U_{D}(kh)+U_{I}(kh)\;k=\text{1},\;\text{2},\;\text{3},\cdots$$ (35)

The term $K_{pi}$ represents the proportional gain for the i-th axis controller, $K_{Di}$ refers to the derivative gain for the i-th axis controller, and $K_{Ii}$ denotes the integral gain for the i-th axis controller.

**Fig 15:**
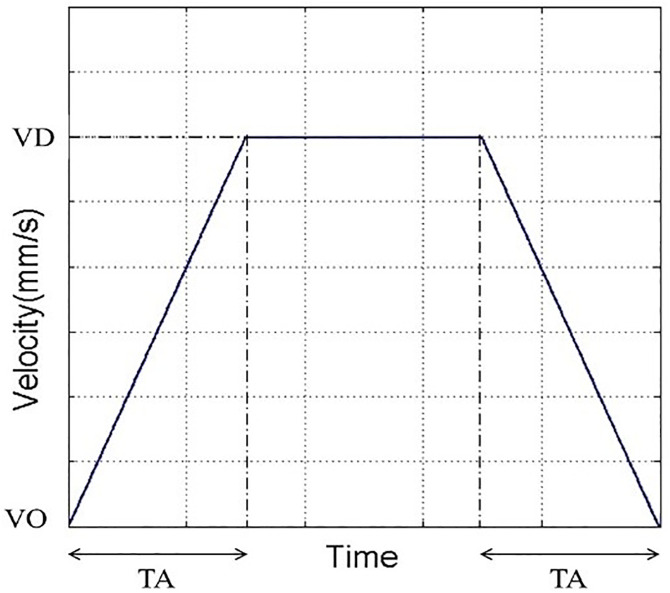
Stepper motor speed trajectory curve.

Based on the XYZ platform structure of the precision positioning stage provided by the manufacturer, the XYZ platform uses stepper motors for precise positioning control [44–45]. To fully leverage the high-speed performance of the stepper motors, the pulse frequency is typically set below the start frequency during startup and then gradually increased to the target speed. The rate of frequency change must ensure that the stepper motor does not skip steps while minimizing the startup acceleration time. To ensure positioning accuracy, the pulse frequency must be gradually reduced before stopping until a stable stopping speed is reached. Therefore, the stepper motor’s operation during high-speed movement and precise positioning involves five stages: “start - acceleration - high-speed operation - deceleration - stop,” with the speed characteristics typically following a trapezoidal distribution. The velocity trajectory curve used in this study is shown in Fig 15, where the initial speed is VO, the acceleration time is TA, and the driving speed is VD.

## 5. Experiment on the precision alignment platform

**Fig 16:**
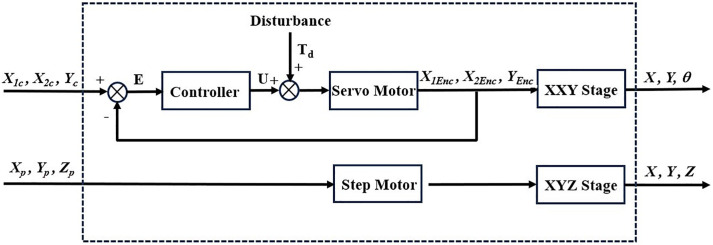
Motion control diagram of the six-axis precision alignment platform.

The motion control block diagram of the six-axis precision alignment platform is shown in Fig 16. The XXY platform employs both PID and MDDS control methods to conduct point-to-point control experiments and trajectory tracking experiments. The results of both control methods are compared to evaluate the effectiveness of the controllers. For the translation and rotation movements of the XXY platform, the motion is ultimately controlled by the actuators of each axis motor. After the mini stage moves, its center point’s translation and rotation can be measured visually, with values for X (mm), Y (mm), and theta (rad). Through inverse kinematics calculations, the corresponding three-axis motor rotation angles, X1 (rad), X2 (rad), and Y (rad), are determined. The XYZ platform then conducts precise positioning control experiments for motor speed trajectories. Finally, through the analysis of experimental data from both the XXY and XYZ platforms, further image positioning control experiments are carried out to verify the overall alignment accuracy and stability of the system.

### 5.1 Point-to-Point control experiment of the XXY platform

In the Point-to-Point (PTP) control experiment of the XXY platform, each axis moves synchronously at a preset speed from the starting point to the target position. Due to differences in movement speed and target distances among the axes, trajectory variations may occur during multi-axis motion. However, all axes ultimately reach the designated target position accurately. The relevant parameters for the XXY platform PTP control experiment are shown in Table 1.

**Table 1:** Experimental control parameters.

Relative parameters	XXY Platform	
**Point-to-Point**	**Trajectory Tracking**
**Initial coordinates (mm, mm, degree)**	[X, Y, Z]= [0, 0,0]	[X, Y, Z]= [0,0,0]
**Target coordinates (mm, mm, degree)**	[X, Y, Z]=[0.5, −0.5, 0.5]	[X, Y, Z] =[0.5, −0.5, 0.5]
**Control sampling rate**	1KHz	1KHz
**Output saturation of each axis**	±10volt	±10volt
**PID control parameters**	K_P1_ = K_P2_ = K_P3_ = 2 K_I1_ = K_I2_ = K_I3_ = 0.0001 K_D1_ = K_D2_ = K_D3_ = 0.008	K_P1_ = K_P2_ = K_P3_ = 2 K_I1_ = K_I2_ = K_I3_ = 0.0001 K_D1_ = K_D2_ = K_D3_ = 0.008
**MDDS control parameters**	*c*_1_ = *c*_2_ = *c*_3_ = 10	*c*_1_ = *c*_2_ = *c*_3_ = 50

**Fig 17:**
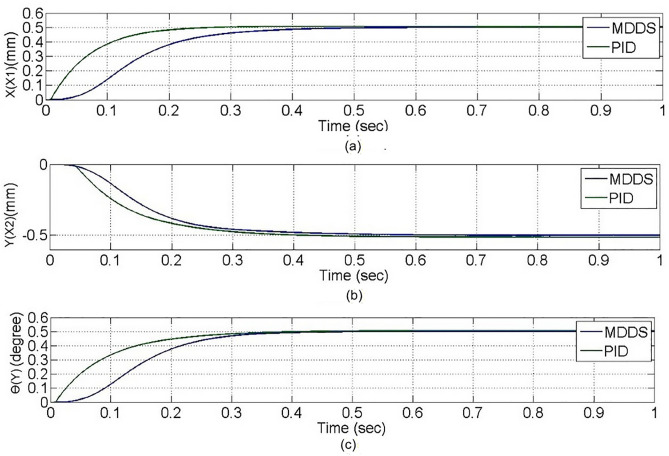
Axial movement trajectory in the Point-to-Point control experiment. **(a)** X1-axis. **(b)** X2-axis. **(c)** Y-axis.

**Fig 18:**
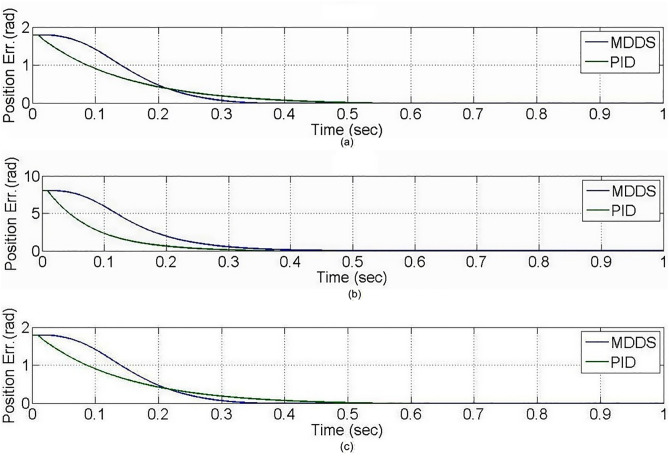
Axial position error in the Point-to-Point control experiment. **(a)** X1-axis. **(b)** X2-axis. **(c)** Y-axis.

In the PTP control experiment, all axes move simultaneously from the initial coordinates to the target coordinates, as shown in Fig 17. From the figure, it can be observed that noticeable differences exist in the motion of each axis at the beginning of the control process; however, after t = 0.3 seconds, all axes reach a stable state and arrive at the target coordinates. The two control methods gradually converge. A more in-depth analysis of the position errors of each axis, using inverse kinematics for conversion, is shown with the motor angles in rad units. According to the experimental results in Fig 18, both the PID and MDDS control methods exhibit larger position errors at the start of the motion, and the errors decrease over time. Between t = 0.3 seconds and t = 0.4 seconds, the position errors of both control methods approach zero, but the MDDS method reaches zero faster than the PID method. For the PID control method, after t = 0.3 seconds, the steady-state error of the X1 axis is within a range of 0.00184 rad, the X2 axis has a steady-state error of 0.00813 rad, and the Y axis has a steady-state error of 0.00266 rad. Using the MDDS control method, after t = 0.3 seconds, the steady-state error of the X1 axis is within 0.00013 rad, the X2 axis error is within 0.00034 rad, and the Y axis error is within 0.00011 rad. Comparing the error ranges of the two control methods, it is evident that the MDDS control method has better control accuracy than the PID control method.

### 5.2. Trajectory tracking control experiment of the XXY platform

**Fig 19:**
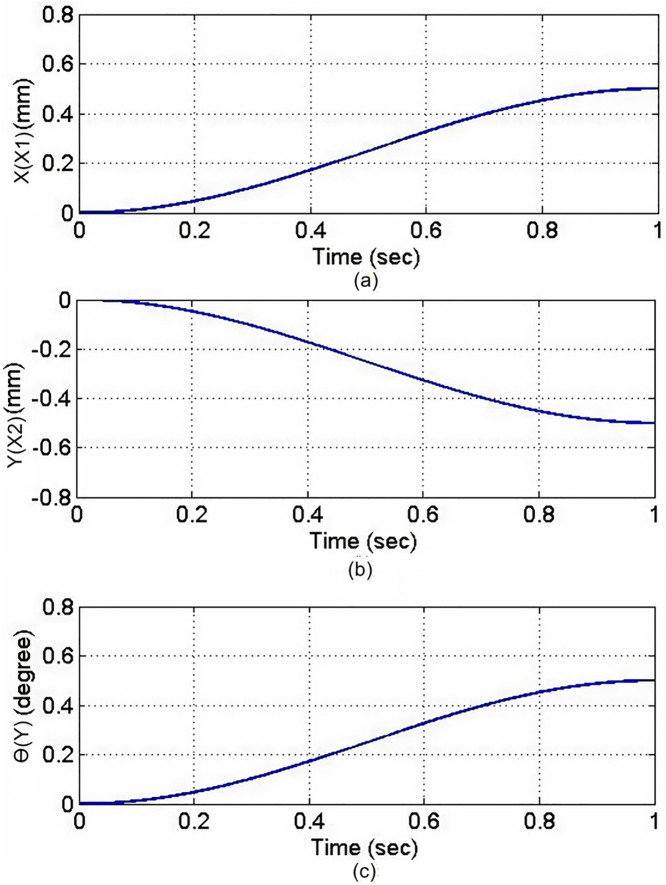
Diagram of each axial movement trajectory. **(a)** X1-axis. **(b)** X2-axis. **(c)** Y-axis.

To further analyze the control performance of PID and MDDS, a linear acceleration and deceleration trajectory tracking experiment was conducted on the XXY platform, as shown in Fig 19. The relevant experimental parameters are listed in Table 1.

**Fig 20:**
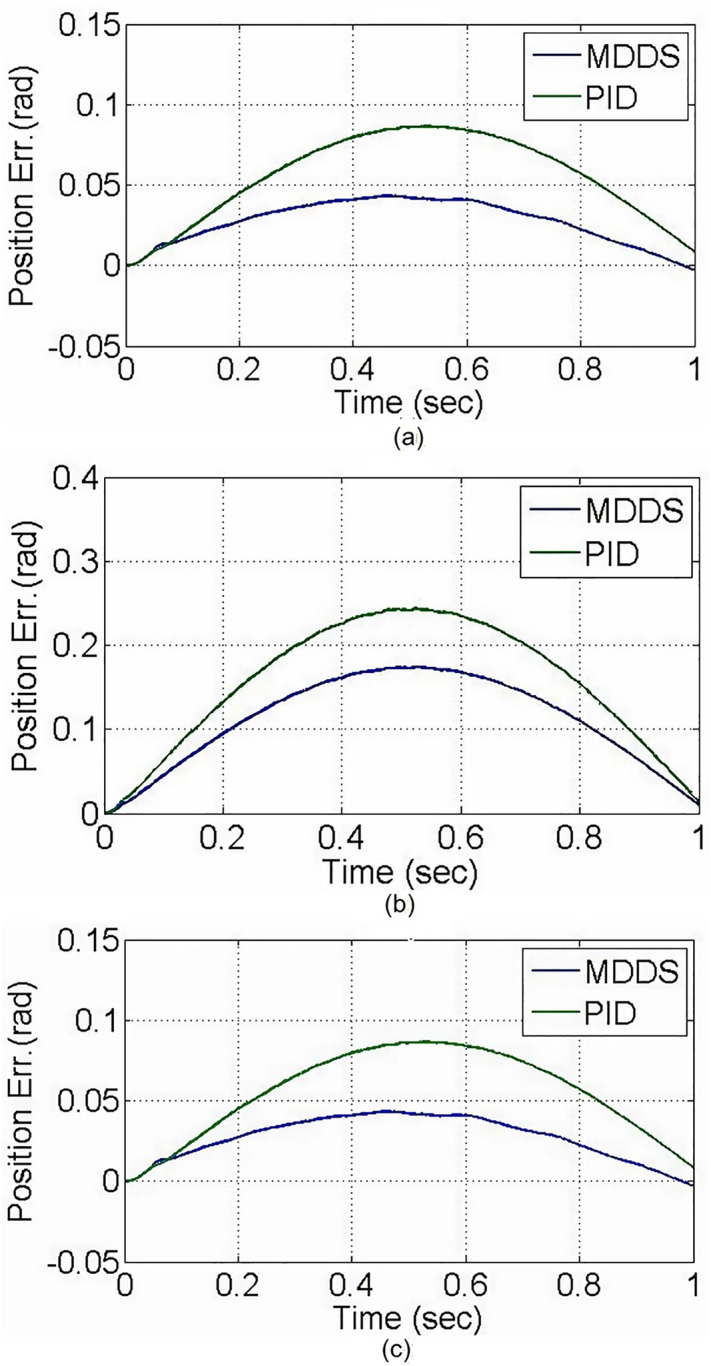
Axial position error in the trajectory tracking control experiment. **(a)** X1-axis. **(b)** X2-axis. **(c)** Y-axis.

The experimental results demonstrate that both PID and MDDS control methods allow each axis to smoothly follow the predefined trajectory. As previously mentioned, the position error of each axis is expressed in terms of motor angle control. During the trajectory tracking process, it is observed that the MDDS control method consistently exhibited smaller position errors compared to PID. For the PID control method, the tracking error range was 0.084 rad for the X1 axis, 0.2411 rad for the X2 axis, and 0.082 rad for the Y axis. In contrast, the MDDS control method achieved a smaller error range of 0.043 rad for the X1 axis, 0.1842 rad for the X2 axis, and 0.045 rad for the Y axis. These results show that MDDS provides superior control precision with reduced error compared to PID, as illustrated in Fig 20.

Also, Figs 18 and 20 illustrate the performance differences between the PID and MDDS control methods across various motion modes. The MDDS strategy allows precise regulation of the system’s response speed through the parameter C_1_ in Table 1, enabling the platform to achieve controlled and stable dynamic behavior in point-to-point motions according to design specifications. In contrast, the PID controller parameters are primarily determined through trial-and-error tuning, which can result in inconsistent response speeds across different motion directions and modes. Consequently, PID may respond faster in certain linear motions but struggles to maintain consistent control performance during rotational movements. MDDS, on the other hand, provides uniform dynamic responses across all motion modes, thereby enhancing overall control stability and motion accuracy.

### 5.3. Motion control experiment of the XYZ platform

In the control experiment of the XYZ platform, stepper motors were employed as the primary actuating components to achieve high-precision positioning and trajectory tracking control. The entire control process was based on pulse control commands issued by the host computer, with each axis synchronously receiving and executing the corresponding pulse signals to realize coordinated multi-axis motion. To ensure smooth motion and dynamic response performance, each actuating axis adopted a trapezoidal velocity curve for acceleration and deceleration control, as shown in Fig 15. This velocity planning method allows smooth transitions between acceleration, constant velocity, and deceleration stages, effectively avoiding mechanical shocks and structural vibrations caused by sudden speed changes, thereby significantly improving the stability and reliability of the XYZ platform during high-speed operation.

**Fig 21:**
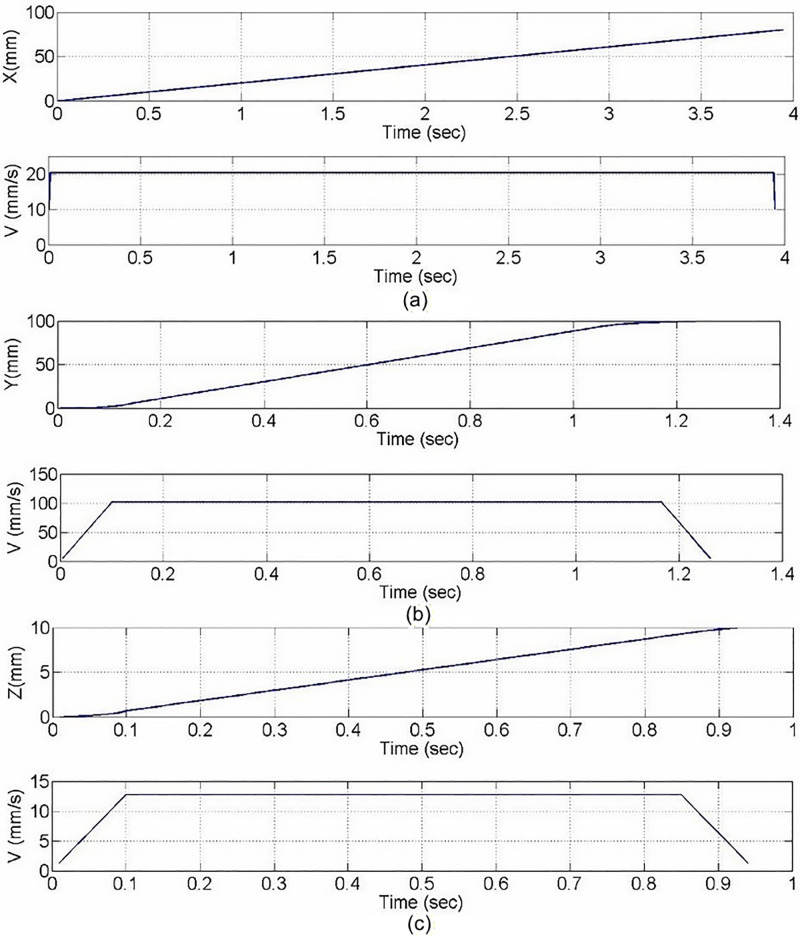
Trajectory and velocity curves of each axis in the XYZ Platform. **(a)** X-axis. **(b)** Y-axis. **(c)** Z-axis.

During the experiment, the initial coordinates of the XYZ platform were set as [X, Y, Z] = [0, 0, 0] (mm, mm, mm), and the target coordinates were [X, Y, Z] = [80, 100,10] (mm, mm, mm). Within this displacement range, each axis achieved smooth acceleration, constant velocity, and deceleration motion under synchronized multi-axis control, exhibiting good trajectory consistency and dynamic coordination. The experimental results, as shown in Fig 21, illustrate the trajectory and velocity variations of each axis. The X-axis velocity was approximately 20 mm/s, the Y-axis velocity was about 100 mm/s, and the Z-axis velocity was around 13 mm/s. The overall motion was stable and free from noticeable vibrations. By analyzing the velocity variations of each axis, it can be observed that the platform achieved smooth transitions during both start-up and stopping phases, demonstrating the practical feasibility and dynamic control effectiveness of the trapezoidal velocity curve approach in the real system.

### 5.4. Image positioning control experiment

**Fig 22:**
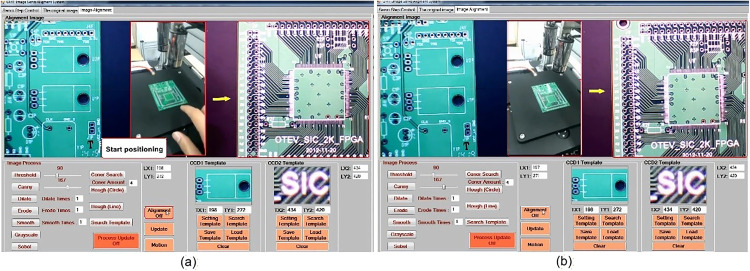
Testing of image-based positioning and human-machine interface. (a) start of positioning. (b) end of positioning.

Before operating the six-axis precision alignment platform, the image positioning technology and the functionality of the HMI (human-machine interface) are tested. A circuit board is placed on the alignment platform, and the system immediately initiated the positioning process upon contact, as shown in Fig 22(a). Within a short period, the alignment is completed successfully, as illustrated in Fig 22(b). As seen in Fig 21, the developed HMI clearly displays the captured image of the circuit board and the positioning outcome, demonstrating the system’s effectiveness in real-time image recognition and precise alignment.

**Fig 23:**
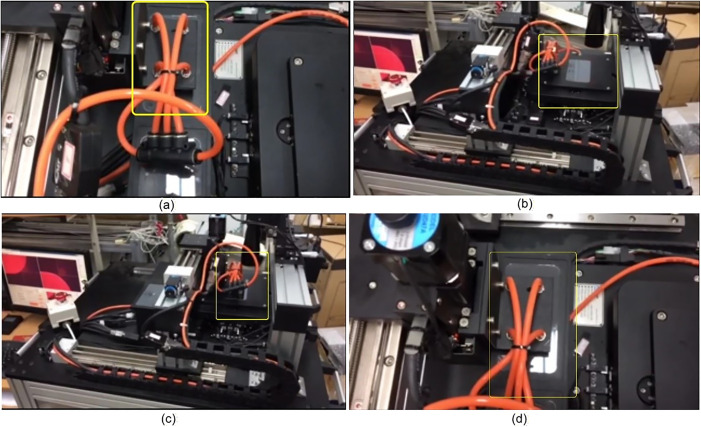
Image-Based Positioning and Motion Control Process of the Six-Axis Precision Alignment Platform from (a) to(d).

Based on the experimental results of the XXY and XYZ platforms, the XXY platform adopts the MDDS control method, and the XYZ platform uses the velocity trajectory curve from the aforementioned experiment. Other relevant experimental parameters are shown in Table 1. As shown in Fig 23, a rectangular glass sheet measuring 125 mm × 65 mm is initially placed flat in the storage area of the platform (Fig 23(a)). Through the control of the XYZ platform, the glass sheet is then transferred to the XXY working platform for alignment (Fig 23(b)). Once the alignment is completed, the XYZ platform retrieves the glass sheet from the XXY platform and returns it to the storage area (Figs 23(c)–23(d)). This process demonstrates the system’s coordination and precision in handling and alignment operations.

**Fig 24:**
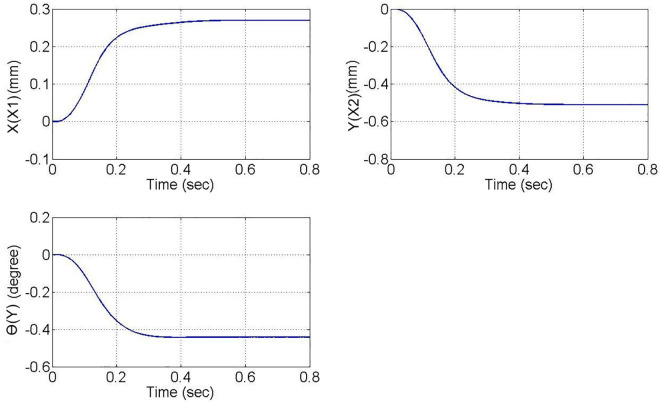
Target Coordinate Diagram.

**Fig 25:**
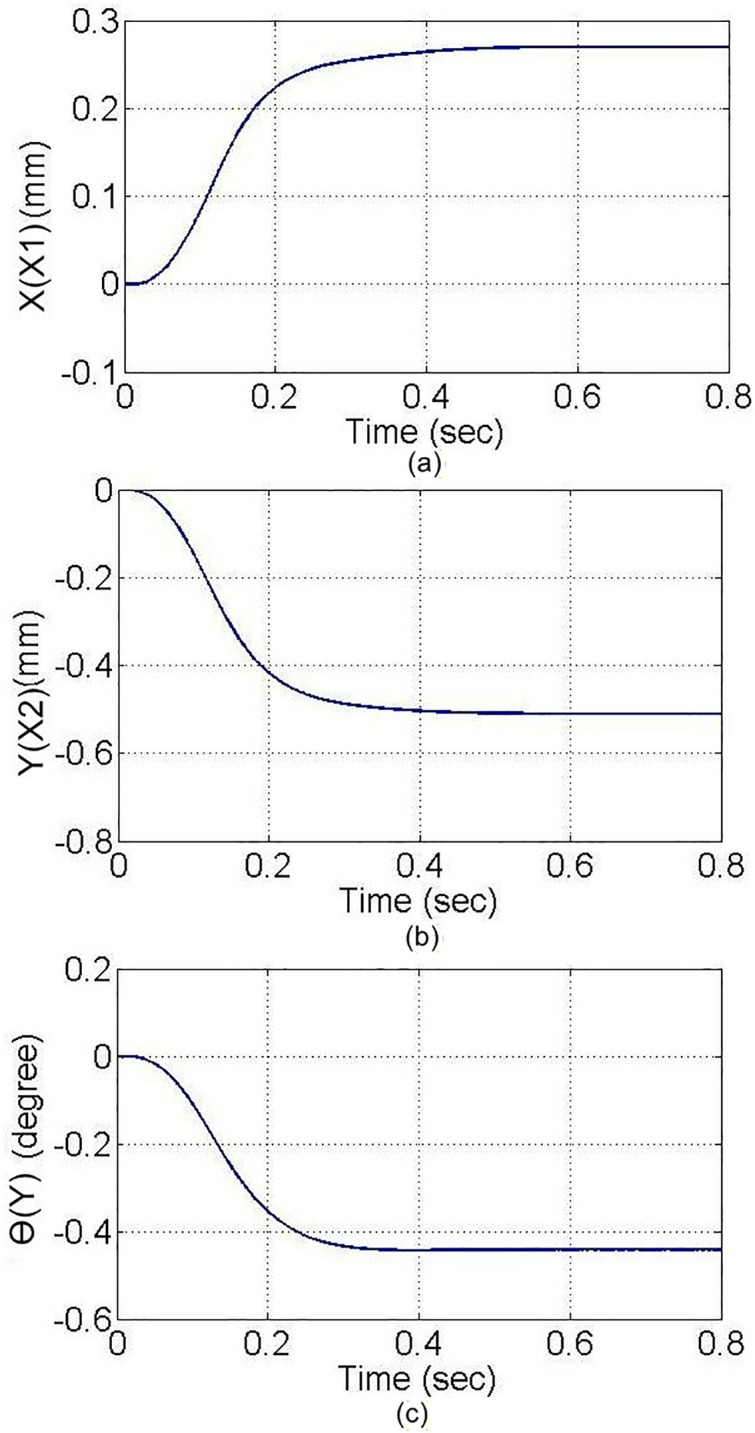
Axial Trajectory Diagram of Image Positioning. **(a)** X1-axis. **(b)** X2-axis. **(c)** Y-axis.

**Fig 26:**
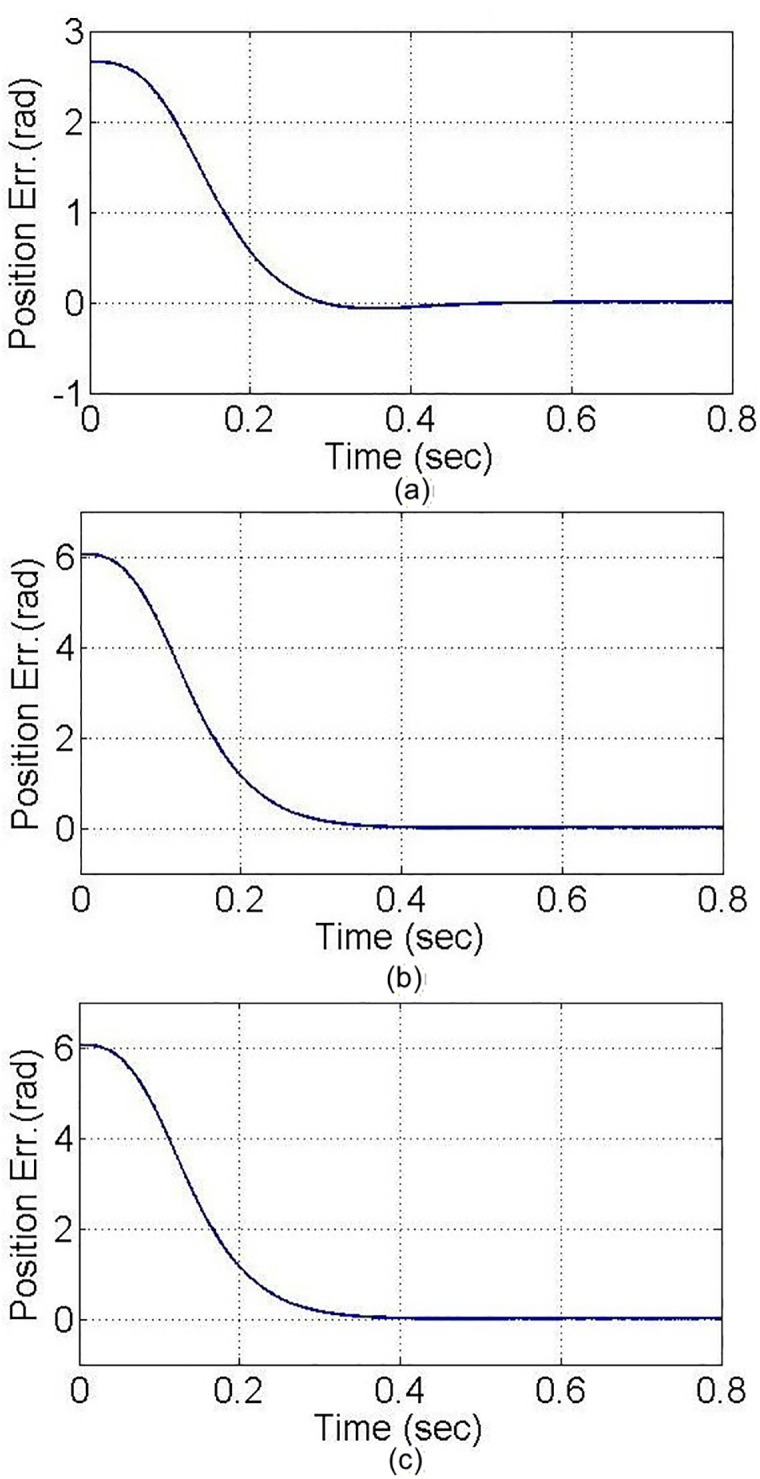
Axial position error in the trajectory tracking control experiment based on image positioning. **(a)** X1-axis. **(b)** X2-axis. **(c)** Y-axis.

A rectangular glass sheet is placed on the workbench. The two CCD image recognition targets are moved in the X direction by 0.26 mm, in the Y direction by −0.5 mm, and at an angle of 0.45 degrees. The two CCDs capture the edge corners of the glass sheet, and the computer processes the image to obtain the target coordinates, calculating the required compensation errors for each actuating axis. The target coordinates are shown in Fig 24. The image recognition speed is approximately 0.1 to 0.3 seconds, with an image recognition error of about 1–2 pixels, and the image recognition accuracy is approximately ±10μm to ±15μm. The axis positioning control trajectories, based on image recognition, are shown in Fig 25. Using the MDDS control method, the axis position errors are represented by motor rotation angles, with the experimental results shown in Fig 26.

From the experimental results in Fig 26, it is evident that under MDDS control, each axis smoothly moves to the target values set by the image positioning results. At the initial stage of movement, there are noticeable position errors; however, these errors gradually decrease over time. After t = 0.3 seconds, the position errors of each axis stabilize. In the steady state, the X1 axis has an error range of 0.00015 rad, the X2 axis has an error range of 0.00082 rad, and the Y axis has an error range of 0.00084 rad. When converted to the displacement of the XXY platform, the movement error is approximately ±0.23 μm, verifying that the image positioning and control method achieve excellent control accuracy and are suitable for high-precision positioning applications.

## 6. Conclusion

This study aims to develop an image-based positioning and control system for a six-axis precision alignment platform by integrating machine vision and image processing technology to achieve precise motion control. A servo stepper control system was constructed using a multi-axis motion control board. To meet the demands of high sampling rates, fast computation, and synchronized multi-axis motion in the control circuit, a Field-Programmable Gate Array (FPGA) was employed as the core, establishing an experimental platform for six-axis precision alignment motion control. In terms of image processing, the system effectively identifies specific object features and positions with high accuracy. Additionally, the target object can be replaced as needed, significantly enhancing the practicality and flexibility of image-based positioning. A human-machine interface (HMI) was designed to facilitate operation, featuring key functionalities such as image recognition-based positioning and motion control. Through these interfaces, users can monitor the operational status of each axis while displaying and storing relevant data. For motion control experiments, a sliding manifold was designed in the state space to serve as the reference for the Manifold Deformation Design Scheme (MDDS) control method, regulating the system’s dynamic response. Experimental results demonstrate that, compared to the traditional PID control method, MDDS exhibits lower tracking errors, higher positioning accuracy, and smoother motion trajectories in point-to-point control, trajectory tracking, and image-based positioning tasks. Moreover, MDDS effectively reduces the complexity of control parameter selection, simplifying the tuning process. In summary, by integrating image processing, FPGA technology, and the MDDS control method, this study successfully achieves high-precision motion control for the six-axis precision alignment platform, providing reliable technological support for applications in the field of precision alignment.
